# Evaluation of A Modified Persian Version of the Developmental Eye Movement Test in Children

**DOI:** 10.18502/jovr.v21.20739

**Published:** 2026-10-14

**Authors:** Enayatollah Osroosh, Saeed Rahmani, Ebrahim Jafarzadehpour, Haleh Kangari, Mohammad Reza Talebnejad, Ali Reza Akbarzadeh Baghban, Saeedeh Hoseinmenni

**Affiliations:** ^1^Department of Optometry, School of Rehabilitation Sciences, Shahid Beheshti University of Medical Sciences, Tehran, Iran; ^2^Department of Optometry, School of Rehabilitation Sciences, Iran University of Medical Sciences, Tehran, Iran; ^3^Poostchi Ophthalmology Research Center, Department of Ophthalmology, School of Medicine, Shiraz University of Medical Sciences, Shiraz, Iran; ^4^Proteomics Research Center, Department of Biostatistics, School of Allied Medical Sciences, Shahid Beheshti University of Medical Sciences, Tehran, Iran

**Keywords:** Developmental Eye Movement Test, Persian Version, Saccadic Eye Movements

## Abstract

**Purpose:**

To adapt and evaluate the Developmental Eye Movement (DEM) test in Persian for Iranian children aged 7-12 years, to evaluate developmental changes across ages, and to compare performance with the American normative sample to identify potential linguistic and cultural influences on oculomotor behavior.

**Methods:**

The DEM test was developed by translating the original numbers into Persian numerals. Awe enrolled a sample of 720 Iranian children aged 7-12 years utilizing a stratified random sampling method from four educational districts in Shiraz. The assessment was administered in accordance with the standardized protocol detailed in the official manual, except that the numerals were read from right to left. To evaluate test reliability, a subset of 160 children aged 8-9 years underwent retesting.

**Results:**

Administration of the DEM test revealed age-related improvements in oculomotor skill that reached a plateau around age 10. Iranian children were slower than the American normative sample on both vertical and horizontal tasks, produced higher ratios, and made more errors in most age groups. These differences had largely evened out by age 12, pointing to the influence of linguistic and orthographic factors. The test was highly reliable across repeated administrations (coefficients: 0.78-0.95).

**Conclusion:**

This study thus provides Persian-language normative data and supports the reliability of the DEM test for this population. This investigation successfully established Persian-language normative data for the DEM test and confirmed the instrument's reliability within an Iranian population. In light of the substantial influence of cultural and linguistic factors on oculomotor behavior, these findings emphasize the critical need to utilize population-specific norms to ensure diagnostic accuracy and prevent the misclassification of typically developing readers.

##  INTRODUCTION

The Developmental Eye Movement (DEM) test is an economical clinical instrument used to assess saccadic eye movement performance and visual–verbal automaticity during reading activities.^[1]^ Deficiencies in oculomotor performance are highly associated with reading problems, and children with such deficiencies often exhibit symptoms such as poor tracking, loss of place, and reduced comprehension.^[2,3,4,5,6,7,8]^ These problems, occurring in children with normal intelligence, may result in school failure and psychosocial disturbances.^[9,10,11]^ Reading disability is the most prevalent type of learning disability, affecting approximately 8-9% of children.^[12,13]^

The DEM test, originally designed for English-speaking populations, provides a number-naming-based quantitative alternative to more complex methods of eye-tracking.^[14,15]^ However, its applicability is presently limited by the lack of language-specific normative data, since it has been substantially sensitive to both linguistic and cultural factors.^[14,15][16][17,18][19]^

Several adaptations of the original DEM test have been developed for different age groups and populations. For example, the Adult Developmental Eye Movement (ADEM) test was first introduced and validated in Spanish-speaking populations using two-digit numbers to increase task difficulty, and was later adapted for English-speaking participants.^[20]^ A further modification, the ADEM, incorporated a number-letter sheet to increase cognitive demand and demonstrated acceptable reliability and internal consistency. These adaptations highlight the importance of modifying and evaluating DEM-based assessments across different linguistic and cultural contexts. In line with these efforts, the current study presents a modified Persian version of the DEM test for Persian-speaking children.

Applying the English normative data to Persian-speaking populations is not appropriate since there are basic orthographic differences, such as the right-to-left reading direction, which influence saccadic eye movement patterns. While French and Hebrew normative data are available, none have been established for Persian-speaking populations.^[21,22]^ Hence, the aim of this study was to develop the first age-specific Persian DEM normative data in children aged 7 to 12 years, determine its test–retest reliability, and finally compare the results with American normative data to improve the identification of oculomotor-related reading disorders among Persian-speaking participants.

##  METHODS 

This cross-sectional study was approved by the ethics committee of Shahid Beheshti University of Medical Sciences and was conducted in accordance with the principles of the Declaration of Helsinki (IR.SBMU.RETECH.REC.1402.877). Written informed consent was obtained from the parents or legal guardians of all participating children, and verbal assent was obtained from the children prior to participation. The consent process included a detailed explanation of the study objectives, procedures, potential risks and benefits, and the voluntary nature of participation. Participants were also informed that they could withdraw from the study at any time without penalty.

The participants consisted of 755 children aged 7-12 years who were initially screened using stratified random sampling from 10 randomly selected primary schools in four educational districts (north, south, east, and west) of Shiraz. The number of selected schools from each district was proportional to the district's student population. District 1 contributed four schools (two girls' and two boys' schools), while Districts 2, 3, and 4 each contributed two schools (one girls' and one boys' school). Within each selected school, 12 children were randomly chosen from each grade (grades 1 through 6), yielding 72 children per school. Sampling was proportionally distributed across grades 1 through 6. The population was then divided into six discrete age groups, each consisting of 120 participants, ensuring an equal distribution of 16.7% across groups. A balanced gender distribution (1:1 ratio) was maintained, and complete demographic data were obtained for all individuals. A single trained optometrist administered the DEM test, and a separate optometrist conducted the optometric screening examinations.

After applying the exclusion criteria, 35 children were removed from the study, 18 were unable to understand the test instructions, and 17 failed to complete the DEM pretest card within the allotted 12-second time limit. The final sample comprised 720 participants. Complete demographic information was obtained for all subjects, and no participant was lost to follow-up after enrollment. A randomly selected subsample of 160 children aged 8-9 years underwent retest reliability assessment within a 2-week interval. Reliability assessment was limited to these age groups because of practical constraints associated with repeated school-based assessments, including scheduling limitations and participant availability within the retest interval. These age groups were selected because they represent the mid-range of the study population and provide a clinically meaningful estimate of the temporal stability of the Persian DEM test. The procedures for participant recruitment, screening, and enrollment are illustrated in Figure 1.

**Figure 1:**
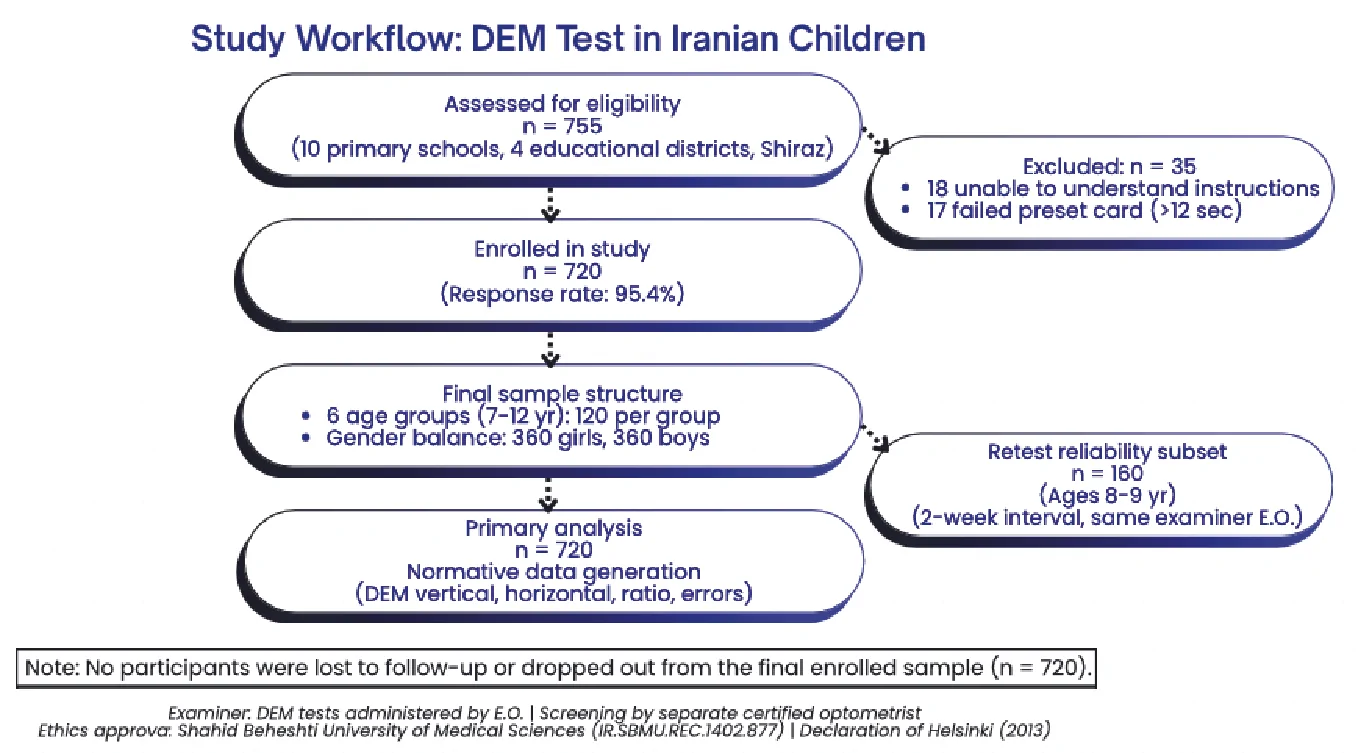
Study flowchart.

The Persian version of the DEM test was developed by preserving the original card layout, spacing, and overall visual structure while replacing the English numerals with Persian numerals. Because Persian and English scripts differ typographically, the exact original font could not be reproduced precisely; therefore, a standard, highly legible Persian font with comparable visual clarity was selected to maintain the intended presentation of the test. The only structural modification involved administering the horizontal subtest from right to left to correspond with Persian reading direction. Although Persian numerals are conventionally read from left to right, the horizontal DEM subtest is intended to evaluate oculomotor scanning behavior during a reading-like task. In Persian readers, habitual text scanning occurs from right to left, and this automatized directional pattern likely influences saccadic behavior even during numeral-naming tasks. Therefore, right-to-left administration better reflects the natural scanning behavior of Persian-speaking children and is consistent with adaptations developed for other right-to-left languages. No additional modifications were made to the test design, scoring system, or structure.

The inclusion criteria were as follows: age between 7 and 12 years; monocular best-corrected distance visual acuity (BCDVA) of 20/25 or better in each eye at 6 meters; near visual acuity of 20/25 or better at 40 cm; absence of clinically significant binocular vision or accommodative anomalies, including normal ocular alignment, phoria within Morgan's criteria, near point of convergence (NPC) ≤ 6 cm, and stereoacuity ≤ 40 arc seconds; successful completion of the DEM pretest card within 12 seconds; and provision of written informed consent from parents or legal guardians. The exclusion criteria included inability to understand test instructions, neurological or physical disabilities, diagnosed dyslexia or other specific learning disabilities, and mental disorders.

To minimize the influence of developmental variation in reading ability and visual–verbal automaticity, we excluded children with diagnosed learning disabilities or dyslexia. Furthermore, the DEM ratio score, which reflects the relationship between horizontal and vertical naming performance, inherently distinguishes visual–verbal automaticity from oculomotor efficiency, thereby reducing the influence of naming speed on the interpretation of oculomotor performance. Completion of the pretest card within 12 seconds additionally confirmed that participants understood the right-to-left scanning requirement and possessed sufficient number-naming automaticity for valid testing.

### Procedure 

Before the DEM test, all participants underwent preliminary visual screenings performed by a certified optometrist, including measurement of monocular best-corrected visual acuity (BCVA) using a Snellen chart at 6 meters under routine indoor lighting conditions. Near visual acuity was assessed at 40 cm using a near Snellen chart. Ocular alignment was evaluated using unilateral and alternating cover tests, and phoria was quantified using a prism bar according to Morgan's norms. NPC was measured using a standard accommodative target moved toward the participant's midline until ocular deviation or diplopia occurred. Stereoacuity was assessed at 40 cm using the Wirt stereopsis test.

The DEM test was administered by a single trained examiner (EO) following the standardized protocol detailed in the official DEM manual. This was achieved using a Persian adaptation of the DEM test (English numerals converted to Persian), with participants reading from right to left to accommodate Persian orthography.^[15]^

Testing was done individually in a quiet, distraction-free room under normal classroom lighting conditions to minimize environmental influences on visual performance and attention. Each child sat comfortably at an individual desk and was asked to hold the test card as they would a book during reading while wearing habitual spectacle correction.^[1]^ Instructions were given for reading the numbers as rapidly and precisely as possible, refraining from using their finger for guidance.

A trained optometrist recorded completion times with a digital stopwatch (HS-80TW, Casio; 0.01-s resolution), starting the timing when the child first vocalized and stopping after the final number was read. All durations were recorded to two decimal places. Participants completed a pretest followed by Cards A, B, and C. Cards A and B each contained 40 vertically arranged numbers, whereas Card C comprised 80 single-digit numerals arranged in 16 rows of 5 numbers each, with randomized spacing between numerals within each row. As isolated single digits, the numerals carry no inherent reading direction. The only adaptation applied was instructing participants to begin each row from the right margin and proceed leftward, consistent with the habitual line-scanning direction of Persian readers. Prior to Card C, all participants completed a pretest card specifically designed to familiarize them with this right-to-left scanning requirement before the timed assessment began. Accuracy was monitored throughout, and four types of error were recorded: transpositions, omissions, substitutions, and additions. Omission occurs when the child skips numbers in the sequence. Addition occurs when the child vocalizes an extra number that is not present on the card. Transpositions occur when two adjacent numbers are read in reverse order, and substitutions involve vocalizing an incorrect number in place of the printed numeral.

The main DEM outcome measures were the vertical score (total time taken for Cards A and B), the horizontal score (completion time for Card C), the adjusted horizontal time (calculated as Card C time × 80 / [80 - omissions + additions]), the ratio score (adjusted horizontal time divided by vertical time), and total errors (sum of all error types).

To assess test–retest reliability, a randomly selected subsample of 160 children aged 8 and 9 years was re-evaluated after a 2-week interval. All repeated assessments were conducted by the same examiner (EO).

### Statistical Analysis 

For each age group, descriptive statistics (means, SDs, 95% CI) were calculated. Children aged 7-12 years were assigned percentile ranks and standardized scores. To aid in the clinical interpretation, percentile ranks and standardized scores were calculated from raw test results. Independent *t*-tests and one-way analysis of variance were used for inferential statistics to observe developmental patterns across age groups, while a paired *t*-test was applied to measure practice effects between test sessions.

In-depth statistical analysis was performed to evaluate the reliability of the DEM test and to uncover age-related performance differences. Intraclass Correlation Coefficient (ICC) was employed to assess reliability in a subsample of 160 participants who were randomly selected from the original 720 children. Test–retest reliability was measured for the five key variables, including vertical time, horizontal time, adjusted horizontal time, ratio, and total errors, and the corresponding 95% CI was reported for each ICC. The whole analysis was performed through SPSS version 25.0 (IBM Corp, Armonk, NY, USA), with statistical significance set at *P* < 0.05. In a one-sample *t*-test, the Persian-speaking children's norms were compared with the English-speaking reference group.

##  RESULTS

The final sample consisted of 720 children (360 girls, 360 boys) aged 7-12 years, recruited from 10 primary schools (5 girls' schools and 5 boys' schools) distributed across four educational districts in Shiraz. The sample was divided into six age groups, each comprising 120 participants with an equal gender distribution (50% boys and 50% girls). Moreover, 35 children were excluded from the initial 755 screened participants.

The DEM test results demonstrated a clear age-related improvement across all parameters. Mean completion times decreased progressively with advancing age. Specifically, vertical time decreased from 69.27 ± 14.42 seconds in the 7-year-old group to 36.41 ± 5.49 seconds in the 12-year-old group, while adjusted horizontal time decreased from 104.21 ± 21.80 to 41.01 ± 7.52 seconds across the same age range. Similar age-related reductions were observed for ratio and total error scores. The comprehensive results are presented as mean ± SD, 95%CI, in Table 1. Figure 2 shows the developmental trend for vertical time (A), adjusted horizontal time (B), error count (C), and ratio (D). One-way ANOVA demonstrated statistically significant age-related differences across all four measures (all *P* < 0.001).

**Table 1:** The DEM test scores for all ages

**Test Age**	**Vertical**		**Adjusted horizontal**		**Ratio**		**Error**	
	**Mean ± SD**	**95% CI**	**Mean ± SD**	**95% CI**	**Mean ± SD**	**95% CI**	**Mean ± SD**	**95% CI**
7-7.11 ${}^{*}$	69.27 ± 14.42	66.66 to 71.88	104.21 ± 21.8	100.27 to 108.15	1.54 ± 0.34	1.48 to 1.60	19.00 ± 8.96	17.39 to 20.61
8-8.11	54.72 ± 10.4	52.84 to 56.60	77.55 ± 17.37	74.41 to 80.69	1.43 ± 0.28	1.38 to 1.49	15.67 ± 8.66	14.10 to 17.23
9-9.11	47.13 ± 8.65	45.57 to 48.70	65.51 ± 14.72	62.85 to 68.17	1.41 ± 0.32	1.35 to 1.47	13.63 ± 8.77	12.05 to 15.22
10-10.11	44.93 ± 8.16	43.46 to 46.41	54.35 ± 10.62	52.43 to 56.28	1.22 ± 0.21	1.18 to 1.26	7.44 ± 6.08	6.35 to 8.54
11-11.11	39.16 ± 5.30	38.17 to 40.16	46.8 ± 9.11	45.15 to 48.44	1.20 ± 0.19	1.16 to 1.23	6.17 ± 5.09	5.25 to 7.09
12-12.11	36.41 ± 5.49	35.31 to 37.30	41.01 ± 7.52	39.65 to 42.37	1.14 ± 0.23	1.10 to 1.18	4.48 ± 4.52	3.67 to 5.30
Total	48.59 ± 14.35	47.54 to 49.64	64.91 ± 25.68	63.03 to 66.78	1.32 ± 0.30	1.30 to 1.35	11.07 ± 8.99	10.41 to 11.72
F	200.417		244.285		42.034		78.153	
*P*	< 0.001		< 0.001		< 0.001		< 0.001	
${}^{*}$ The age range is 7 years to 7 years and 11 months.								

**Figure 2:**
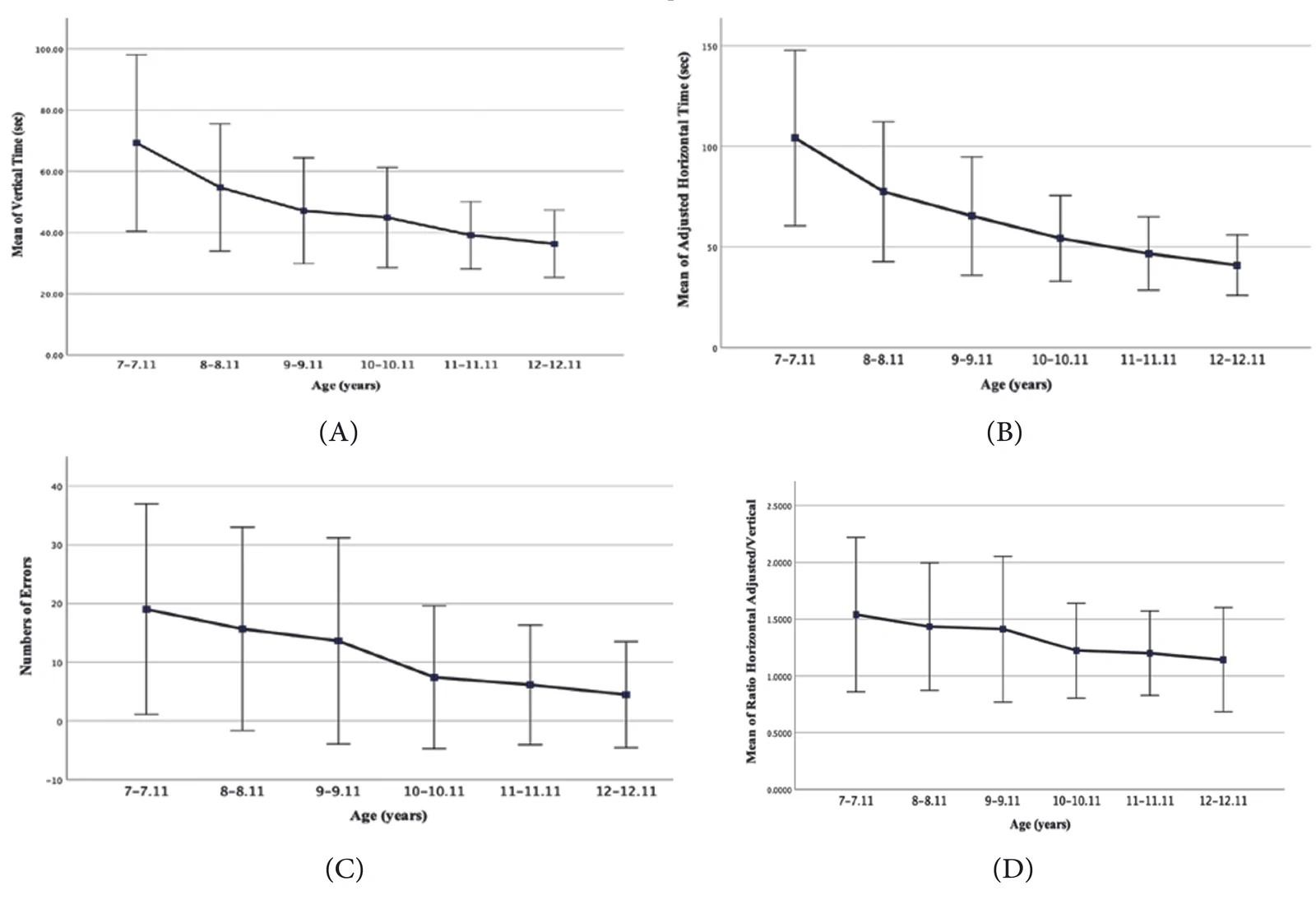
Vertical time (A), adjusted horizontal time (B), errors (C), and ratio (D) of the developmental eye movement subtests for each age group.

### Reliability of the DEM test 

The test–retest reliability of the Persian DEM test was assessed by calculating the ICC in a subsample of 160 children aged 8 and 9 years who underwent repeat testing after a 2‑week interval by the same examiner (EO). Reliability analysis was limited to the 8- and 9-year-old groups due to practical constraints during school-based data collection. Table 2 presents ICC values and their corresponding 95% CIs for the following key parameters: vertical time, adjusted horizontal time, ratio, and total errors, stratified by age (8 and 9 years) and gender. Data are presented separately for boys and girls in each age group, with a final row reporting overall ICC values for the full retest subsample (n = 160).

**Table 2:** ICC coefficients for test–retest comparisons

**Gender**	**Age**	**Vertical**			**Adjusted horizontal**			**Ratio**			**Total errors**		
		**ICC**	**95% CI**		**ICC**	**95% CI**		**ICC**	**95% CI**		**ICC**	**95% CI**	
		**value**	**Lower band**	**Upper band**	**value**	**Lower band**	**Upper band**	**value**	**Lower band**	**Upper band**	**value**	**Lower band**	**Upper band**
Male	8-8.11	0.87	0.75	0.93	0.91	0.84	0.95	0.6	0.25	0.79	0.82	0.67	0.9
	9-9.11	0.79	0.61	0.89	0.85	0.71	0.92	0.61	0.27	0.79	0.79	0.61	0.89
Female	8-8.11	0.88	0.77	0.93	0.86	0.73	0.92	0.69	0.42	0.84	0.76	0.56	0.78
	9-9.11	0.83	0.69	0.9	0.87	0.76	0.93	0.78	0.59	0.88	0.83	0.68	0.91
Total		0.88	0.84	0.91	0.88	0.84	0.91	0.68	0.57	0.77	0.81	0.75	0.86
ICC, intracluster correlation; CI, confidence interval													

This study compared the mean DEM test scores obtained from Persian-speaking children with those of the English-speaking normative reference group using a one-sample *t-*test. Table 3 compares DEM indices between Persian-speaking Iranian children (*n* = 720) and the American normative sample reported by Garzia and Richman (1990). Vertical time was significantly longer in the Iranian sample across all age groups, with mean differences ranging from 1.27 to 14.44 seconds (all *P-values* ≤ 0.02). Similarly, adjusted horizontal time was significantly higher among Iranian children at all ages, with differences ranging from 1.66 to 19.82 seconds (all *P* ≤ 0.017). For the ratio parameter, Iranian children demonstrated significantly higher values than American children at ages 8, 9, and 11 (all *P-values* < 0.001). No statistically significant differences were observed at ages 10 and 12 (*P* = 0.08 and *P* = 0.296, respectively). At age 7, the Iranian group showed a slightly lower ratio value compared with the American sample, with borderline statistical significance (*P* = 0.05). Total error scores were significantly higher in the Iranian sample across all age groups, with mean differences ranging from 3.37 to 11.46 errors (all *P*-values < 0.001). Detailed mean values and standard deviations for both populations are presented in Table 3.

**Table 3:** Comparison of differences in the DEM test indices in Iranian Persian-speaking and American English-speaking children

**Age**	**Parameter**	**Iranianmean (SD)**	**Americanmean (SD)**	**Meandifference**	***P*** **-value**
7	Vertical time	69.27 (14.42)	54.83 (9.20)	14.44	< 0.001
	Adjusted Horizontal	104.21 (21.80)	87.94 (28.18)	16.27	< 0.001
	Ratio	1.54 (0.34)	1.60 (0.41)	–0.06	0.05
	Error	19.00 (8.96)	12.50 (12.91)	6.50	< 0.001
8	Vertical time	54.72 (10.40)	46.76 (7.89)	7.96	< 0.001
	Adjusted Horizontal	77.55 (17.37)	57.73 (12.32)	19.82	< 0.001
	Ratio	1.43 (0.28)	1.24 (0.18)	0.19	< 0.001
	Error	15.67 (8.66)	4.61 (6.91)	11.06	< 0.001
9	Vertical time	47.13 (8.65)	42.33 (8.20)	4.80	< 0.001
	Adjusted Horizontal	65.51 (14.72)	51.13 (13.30)	14.38	< 0.001
	Ratio	1.41 (0.32)	1.21 (0.19)	0.20	< 0.001
	Error	13.63 (8.77)	2.17 (4.10)	11.46	< 0.001
10	Vertical time	44.93 (8.16)	40.28 (4.73)	4.65	< 0.001
	Adjusted Horizontal	54.35 (10.62)	47.64 (10.11)	6.71	< 0.001
	Ratio	1.22 (0.21)	1.19 (0.17)	0.03	0.08
	Error	7.44 (6.08)	1.91 (2.68)	5.53	< 0.001
11	Vertical time	39.16 (5.30)	37.14 (5.42)	2.02	< 0.001
	Adjusted Horizontal	46.80 (9.11)	42.62 (7.61)	4.18	< 0.001
	Ratio	1.20 (0.19)	1.15 (0.13)	0.05	0.005
	Error	6.17 (5.09)	1.68 (2.34)	4.49	< 0.001
12	Vertical time	36.41 (5.49)	35.14 (5.87)	1.27	0.02
	Adjusted Horizontal	41.01 (7.52)	39.35 (8.11)	1.66	0.017
	Ratio	1.14 (0.23)	1.13 (0.10)	0.01	0.296
	Error	4.48 (4.52)	1.11 (1.17)	3.37	< 0.001
DEM, developmental eye movement; SD, standard deviation					

##  DISCUSSION 

This study presents the first normative adaptation of the DEM test for Persian-speaking children. The findings suggest that DEM scores improve with age, with the most pronounced changes observed in younger age groups, reflecting the developmental maturation of oculomotor control and visual–verbal automaticity. Nevertheless, the Persian DEM showed acceptable test–retest stability and internal consistency, confirming its feasibility as a practical screening tool for reading-related oculomotor function. Systematic performance differences compared to English-speaking norms attest to the importance of orthography, script directionality, and educational context, emphasizing the need for local normative data.

This study employed a cross-sectional design in which all participants were assessed at a single time point and stratified by age. Comparisons across age groups reflect between-group developmental differences rather than longitudinal change. Similarly, comparisons with American normative values were secondary cross-cultural analyses based on published reference data and do not alter the study's cross-sectional nature.

The age-associated improvements in DEM performance reflect the established principles of visuomotor development. The time taken for completion to decrease with age is indicative of the maturation of neural pathways governing eye movements and automaticity.^[19,23]^ Notably, vertical times plateaued earlier than horizontal and adjusted horizontal times, which continued to improve, suggesting the latter metrics are more sensitive indicators of developmental progression. The relative stability of ratio scores and error counts across older age groups suggests that different aspects of oculomotor control mature at varying rates. Such behaviorally linked developments are in line with known neurodevelopmental changes in the frontoparietal, frontostriatal, and corticocerebellar systems that continue into adolescence.^[24,25]^ The plateau reached by both Persian and English groups by age 10—before early adolescence—demonstrates functional maturation of oculomotor systems, while persisting differences in errors indicate language-specific processing demands.^[26,27,28]^

Attention and visuospatial skills are important contributing factors in DEM performance.^[29,30]^ Although some might attribute lower scores to fatigue, the consistently stable error rates in this and previous studies indicate that sustained attention is likely the main factor influencing accuracy.^[1,31,32]^ Research points to the involvement of attentional networks, including the parietal cortex and superior colliculus, in modulating saccadic speed and accuracy.^[33,34,35,36]^ Behavioral patterns such as impulsive responding and delayed error correction further highlight the cognitive processes that contribute to DEM performance.^[16]^

The Persian DEM was highly reliable in terms of ICCs. The results showed good test–retest reliability (0.78-0.95) for vertical time, adjusted horizontal time, and error variables. Meanwhile, the ratio variable showed moderate reliability, consistent with previous reports of instability in composite indices.^[37]^ The outcomes of the Persian version of the DEMs are generally comparable to prior international validation studies, including the Chinese and American versions.^[19,38]^

Cross-cultural comparisons seem to uncover developmental differences. The Persian-speaking children were slower and made more errors than the English-speaking children in almost every age group studied, with significant differences in the ratio at ages 8, 9, and 11 years. These differences can be attributed to the interaction of linguistic, orthographic, educational, and sociocultural variables.^[15]^ It is notable that the right-to-left direction of Persian script and the later onset of formal reading instruction in Iran likely influence saccadic planning and visual–verbal automaticity, consistent with patterns observed in other non–left-to-right orthographies.^[39,40,41]^ The persistence of significant differences in error patterns through age 12 years suggests that language-specific processing demands continue to shape reading-related oculomotor behavior beyond the period of basic visuomotor maturation.

The strong reliability and age-related trajectory observed in this study mirror the findings from other language adaptations, confirming the universal nature of basic oculomotor maturation.^[15,16,19]^ However, significant cross-linguistic differences demonstrate the critical limitation of applying foreign normative data and extend the literature by providing established norms for a right-to-left script population.^[17,18]^ The persistence of differences in error patterns suggests that theoretical models of reading-related oculomotor development have to consider and explain the language-specific determinants of speed and accuracy.^[17,18][19]^

This study is characterized by a large, age-stratified sample and strong psychometric properties, contributing to filling a critical gap for Persian orthography. Limitations include the lack of direct oculomotor measurement (such as eye-tracking), incomplete sociodemographic information, and the tentative attribution of differences to script directionality without direct causal evidence. Learning effects were minimized in the normative sample (*n* = 720) by using a cross-sectional design in which each participant completed the DEM only once, thereby preventing repeated exposure from influencing age-related comparisons. In the test–retest reliability subsample (*n* = 160), a 2-week interval was implemented to reduce short-term memory retention of specific number sequences, and standardized instructions were maintained across administrations. Nevertheless, because no alternate DEM form was available in the reliability subsample, practice effects cannot be completely excluded and should be considered when interpreting the reliability estimates.

The adapted DEM with local norms offers a valid, evidence-based screening tool to identify Persian-speaking children who may be qualified for comprehensive reading and visual assessment. It supports the incorporation of vision screening into the educational framework, with the understanding that a culturally normed cut-off score may be applied. Thus, these findings suggest the importance of local norming for cross-cultural psychometric tools and recommend multimodal assessment methods instead of relying on only one measure. The test provides a practical, inexpensive means of identifying the visual factors contributing to reading difficulties. As such, its implementation could help standardize vision screenings and develop informed interventions within the healthcare and educational systems of Iran.

Understanding DEM performance across development requires models that integrate the combined effects of naming speed, orthographic familiarity, and reading-direction habits, particularly when interpreting patterns of automaticity and accuracy. While the DEM is a useful clinical tool in assessing functional reading performance, its precise relationship to specific saccadic patterns remains to be confirmed through further research. The causal relevance of specific linguistic factors remains quite difficult to establish without direct mechanistic exploration of performance patterns. This study underlines a rather complex relationship between visual processing and linguistic factors, rendering the DEM an important measure of reading-related oculomotor skills.

Future research should include eye-tracking and standardized reading assessments to establish direct explanatory connections. Improving theoretical models and strengthening the clinical accuracy of the DEM will require better demographic controls, closer examination of cognitive factors such as impulsivity and metacognitive monitoring, and the use of more comprehensive multimodal assessment approaches. Further multidisciplinary research is warranted to fully understand the cognitive mechanisms involved and ensure that the test works equally well across different socioeconomic groups. Establishing reliable, language-specific normative data is crucial, and deploying local benchmarks rather than foreign norms is essential for making accurate diagnostic decisions in Persian-speaking populations.

##  Financial Support and Sponsorship

None.

##  Conflicts of Interest

None.
